# Conventional Co-Housing Modulates Murine Gut Microbiota and Hematopoietic Gene Expression

**DOI:** 10.3390/ijms21176143

**Published:** 2020-08-26

**Authors:** Jichun Chen, Shuling Zhang, Xingmin Feng, Zhijie Wu, Wendy Dubois, Vishal Thovarai, Sonia Ahluwalia, Shouguo Gao, Jinguo Chen, Tyler Peat, Shurjo K. Sen, Giorgio Trinchieri, Neal S. Young, Beverly A. Mock

**Affiliations:** 1 Hematology Branch, National Heart, Lung, and Blood Institute, National Institutes of Health, Bethesda, MD 20892, USA; chenji@nhlbi.nih.gov (J.C.); fengx2@nhlbi.nih.gov (X.F.); wuz5@nhlbi.nih.gov (Z.W.); gaos2@nhlbi.nih.gov (S.G.); 2 Laboratory of Cancer Biology and Genetics, National Cancer Institute, National Institutes of Health, Bethesda, MD 20892, USA; zhangsh@nci.nih.gov (S.Z.); duboiswe@mail.nih.gov (W.D.); peattj@nci.nih.gov (T.P.); 3 Basic Science Program, Frederick National Laboratory for Cancer Research, Frederick, MD 21702, USA; thovaraivv@nci.nih.gov (V.T.); soahluwalia@gmail.com (S.A.); sensh@nci.nih.gov (S.K.S.); 4 Center for Human Immunology and Autoimmunity, National Institutes of Health, Bethesda, MD 20892, USA; chenjinguo@mail.nih.gov; 5 Cancer and Inflammation Program, Center for Cancer Research, National Cancer Institute, National Institutes of Health, Bethesda, MD 20892, USA; trinchig@nci.nih.gov

**Keywords:** mice, specific pathogen free, conventional co-housing, gut microbiota, single cell RNA-seq, hematopoietic stem and progenitor cells

## Abstract

Specific-pathogen-free (SPF) mice have improved hematopoietic characteristics relative to germ-free mice, however, it is not clear whether improvements in hematopoietic traits will continue when the level of microorganism exposure is further increased. We co-housed SPF C57BL/6 mice in a conventional facility (CVT) and found a significant increase in gut microbiota diversity along with increased levels of myeloid cells and T cells, especially effector memory T cells. Through single cell RNA sequencing of sorted KL (c-Kit^+^Lin^−^) cells, we imputed a decline in long-term hematopoietic stem cells and an increase in granulocyte-monocyte progenitors in CVT mice with up-regulation of genes associated with cell survival. Bone marrow transplantation through competitive repopulation revealed a significant increase in KSL (c-Kit^+^Sca-1^+^Lin^−^) cell reconstitution in recipients of CVT donor cells which occurred when donors were co-housed for both one and twelve months. However, there was minimal to no gain in mature blood cell engraftment in recipients of CVT donor cells relative to those receiving SPF donor cells. We conclude that co-housing SPF mice with mice born in a conventional facility increased gut microbiota diversity, augmented myeloid cell production and T cell activation, stimulated KSL cell reconstitution, and altered hematopoietic gene expression.

## 1. Introduction

Laboratory mice have been used extensively as model animals for the study of human diseases, but the standard specific-pathogen-free (SPF) housing environment causes the immune system of laboratory mice to be insufficiently trained, due to exposure to only a very narrow spectrum of microorganisms [1]. Mice living in the wild have much broad gut microbiota diversity than do laboratory mice in SPF quarters [2]; “Wilding” mice, created by transferring embryos from laboratory mice into wild mice, have natural microbiota that have outcompeted the microbiota in laboratory microbiota, and are more resilient in their response to environment challenges [3]. Gut microorganisms form a special community capable of regulating a large array of functional activities in humans and mice [4,5]; whereby diversified microbiota can have health benefits by maintaining hematopoiesis [6,7], regulating immunity [1,2], controlling inflammation and infection [8,9], and enhancing cancer treatments [10].

For hematopoietic and immune functions, microbiota diversity has been associated with increased ability of bone marrow (BM) cells to form robust hematopoietic colonies when comparing germ-free (GF) with SPF mice [6,11,12]. Antibiotic treatment to deplete intestinal microbiota impairs the recovery of lymphocytes and neutrophils during syngeneic BM transplantation [13]. In patients receiving allogeneic transplantation of hematopoietic stem and progenitor cells (HSPCs), high intestinal bacterial diversity has been associated with better overall survival [14]. Indirect modulation of gut microbiota can alter the risk of acute graft-versus-host disease [15]. In contrast, antimicrobial treatment to patients with cirrhosis has been associated with thrombocytopenia, anemia and marrow hypoplasia [16].

We employ laboratory mice as model animals to study pathophysiology of human diseases. We were particularly interested in a co-housing approach that was reported to enable normal laboratory mice to gain exposure to a broader spectrum of microorganisms. Recapitulation of adult human immune traits in laboratory mice following co-housing with pet-store mice provided strong evidence showing that restoring physiological microbial exposure in laboratory mice could better model immunological events in humans [1]. Previously, we found that mice born and raised in a conventional facility (CVB) exert greater ability to suppress foreign T cell activation and to attenuate immune-mediated BM destruction than SPF-housed mice [17]. As such, we reasoned that a partial enhancement in microbial diversity by exposing laboratory mice to a “conventional” living environment might also augment immune and hematopoietic traits to better serve as models for the study of human diseases. Thus, we adapted the co-housing method by transferring C57BL/6 (B6) mice from their normal SPF environment to a conventional housing facility (CVT) and housed these CVT with CVB mice. Conventional co-housing enriched gut microbial biodiversity, increased innate and acquired immune cellular immunity, and altered HSPC cellular composition, gene expression and functional characteristics.

## 2. Results

### 2.1. Increased Blood Leukocytes and Activated T Cells in Co-Housed CVT Mice

**Figure 1:**
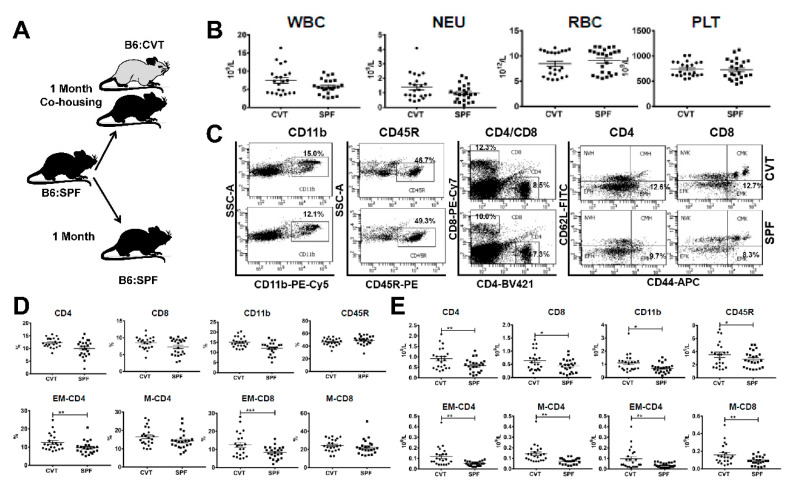
(**A**) C57BL/6J (B6) mice born and raised in specific-pathogen-free (SPF) conditions were either maintained in SPF or transferred to a conventional facility and co-housed (CVT) with mice born and raised in that facility for one month. (**B**) CVT (n = 22) and SPF (n = 23) mice were bled at one month of co-housing to analyze concentrations of white blood cells (WBC), neutrophils (NEU), red blood cells (RBC) and platelets (PLT). (**C**) Blood leukocytes were stained with specific antibodies and were analyzed by flow cytometry for the proportions of myeloid cells (CD11b), B cells (CD45R), helper T cells (CD4), and cytotoxic T cells (CD8), while CD4 and CD8 T cells were further divided, based on expression of CD62L and CD44, into naive (NV, CD44^−^CD62L^+^), central memory (CM, CD44^+^CD62L^+^), effector (EF, CD44^−^CD62L^−^) and effector memory (EM, CD44^+^CD62L^−^) subsets, all shown as representative dot plots. (**D**) Proportions of CD4, CD8, CD11b, CD45R, EM-CD4, M-CD4 (memory CD4, combined CM-CD4 and EM-CD4), EM-CD8 and M-CD8 (memory CD8, combined CM-CD8 and EM-CD8) cells in peripheral blood of CVT and SPF mice. (**E**) Calculated concentrations of CD4, CD8, CD11b, CD45R, EM-CD4, M-CD4, EM-CD8 and M-CD8 cells in peripheral blood of CVT and SPF mice. Data were combined from two experiments performed at different times. * *p* < 0.05; ** *p* < 0.01; *** *p* < 0.001.

After one month of co-housing (Figure 1A), CVT mice appeared normal without significant change in white blood cells (WBC), neutrophils (NEU), red blood cells (RBC), and platelets (PLT) relative to SPF mice maintained under SPF conditions (Figure 1B). We stained blood samples for flow cytometry analyses and divided blood leukocytes into CD4 T cells, CD8 T cells, CD11b myeloid cells and CD45R B cells (Figure 1C). CD4 and CD8 T cells were further divided into naive (NV, CD44^−^CD62L^+^), central memory (CM, CD44^+^CD62L^+^), effector (EF, CD44^−^CD62L^−^), and effector memory (EM, CD44^+^CD62L^−^) subsets based on CD44 and CD62L expression (Figure 1C). While proportions of CD4, CD8 and CD11b cells were only slightly higher in CVT mice, proportions of both EM-CD4 and EM-CD8 T cells were significantly higher in CVT mice (Figure 1D). We calculated cellular concentrations based on proportions and total WBC counts and found significantly increased levels of CD4, CD8, CD11b, CD45R, EM-CD4 and EM-CD8 cells in circulating blood in CVT mice relative to those in SPF mice (Figure 1E). Memory CD4 (M-CD4) and memory CD8 (M-CD8) T cells, computed by combining CM and EM cell fractions, were also significantly increased in CVT mice relative to SPF mice (Figure 1E).

### 2.2. Enrichment of Gut Microbiota Diversity in CVT Mice

**Figure 2:**
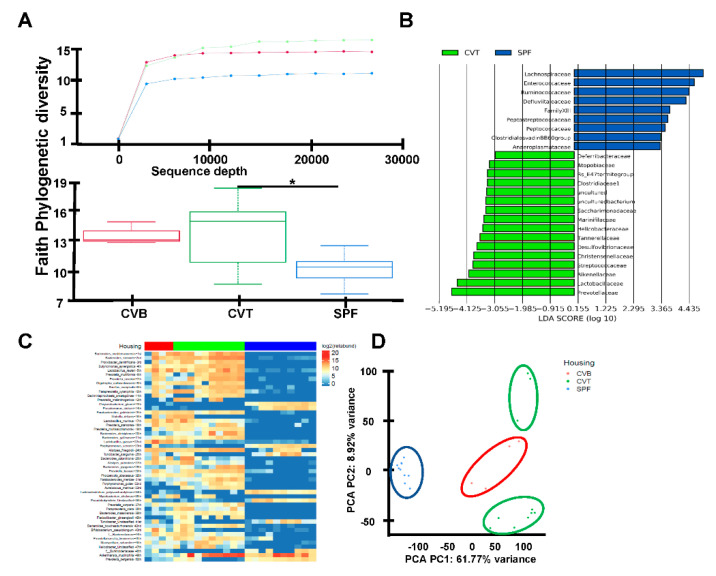
(**A**) C57BL/6J (B6) mice born and raised in specific-pathogen-free (SPF) facilities were either maintained in SPF or were transferred to a conventional facility and co-housed (CVT) with mice born in that facility (CVB) for one month. Fecal samples were collected from SPF (n = 18), CVB (n = 3), and CVT (n = 15) mice at one (n = 12) or six to twelve (n = 3) months of co-housing, and were then processed for DNA extraction and 16S rRNA gene amplicon sequencing to assess microbiota phylogenetic diversity, shown as rarefaction plot using the Faith phylogenetic diversity metric for alpha-diversity and box plots showing significant difference (*p* value = 0.01) in Faith Phylogenetic diversity between CVT and SPF mice. (**B**) Differentially abundant taxa across CVT and SPF mice are shown as LEFse plot. (**C**) Fecal DNA samples from CVT (n = 8), SPF (n = 9) and CVB (n = 4) mice were also proceeded for shotgun metagenomics analyses shown as ranked 50 most variant last known taxa differentially represented in CVT, SPF and CVB mice. (**D**) Display of taxa data based principal components 1 and 2 distribution resulted in specific clusters for CVT, SPF and CVB fecal samples.

These increases in EM-CD4, EM-CD8, M-CD4 and M-CD8 T cells in CVT mice were similar to observations from previously reported pet-store co-housed mice, and we hypothesized that conventional co-housing altered mouse gut microbiota as had housing with pet-store mice [1]. To address this possibility, we first compared the gut flora of CVT and SPF using 16S rRNA amplicon sequencing of fecal samples. CVT mice had a broader spectrum of gut microbials relative to SPF mice, shown as alpha diversity rarefaction curves (Figure 2A), using the Faith Phylogenetic Distance metric [18]; box plots demonstrate phylogenetic diversity in CVT mice relative to SPF mice (Figure 2A). Comparisons between CVT and SPF mice revealed distinctive microbiotic ecosystems: CVT mice had higher representations of sixteen operational taxonomic units (OTU) led by *Prevotellaceae, Lactobacillaceae* and *Rikenellaceae*, while SPF mice had higher representation of nine OTUs led by *Lachnospiraceae, Enterococcaceae* and *Ruminococcaceae* (Figure 2B). Additional analyses of microbiota diversity utilized shotgun metagenomic sequencing of 24 fecal samples and identified the top 50 taxa (primarily at the species level) differentially represented among the SPF, CVT and CVB mice (Figure 2C). Of the top fifteen differentially represented species thirteen had a higher level of representation in CVT than in SPF mice (Table A1). Principal component analysis determined that SPF samples formed a cluster clearly distinct from CVT and CVB samples (Figure 2D), indicating effective transfer of microbiota from CVB to CVT mice through co-housing.

### 2.3. Gene Expression in KL Cells by Single Cell RNA-Seq

**Figure 3:**
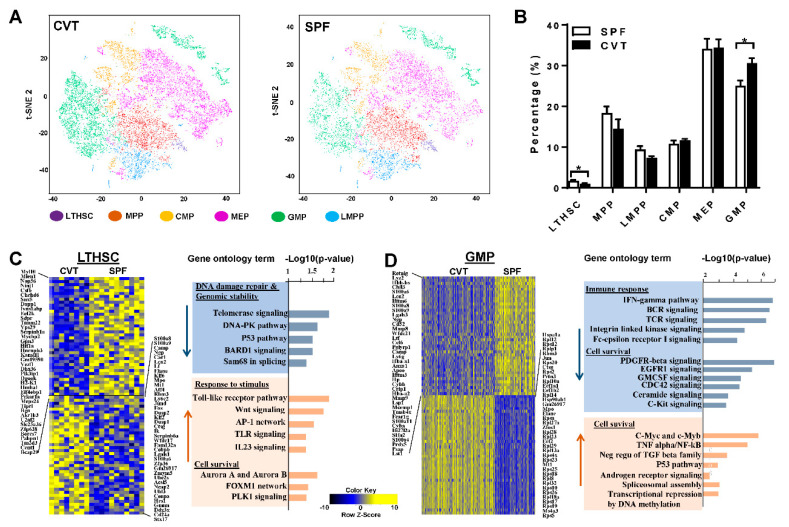
(**A**) BM cells from CVT (n = 8) and SPF (n = 8) mice at one month of co-housing were sorted to collect KL (Kit^+^Lin^−^) cells, pooled to form 4 KL cell pools for each housing condition, and then analyzed by single-cell RNA-seq. t-distributed stochastic neighbor embedding (tSNE) plot revealing KL single-cell gene expression in various hematopoietic stem and progenitor cell (HSPC) subsets in CVT and SPF mice: LTHSC, long-term hematopoietic stem cells; MPP, Multipotent progenitors; CMP, common myeloid progenitors; MEP, megakaryocytic-erythroid progenitors; GMP, granulocyte-monocyte progenitors; LMPP, lymphoid-primed multipotent progenitors. (**B**) Percentages of HSPC subsets in KL cells from CVT and SPF mice. (**C**) Gene expression heatmap and pathway analyses for LTHSCs showing up- and down-regulation of genes in CVT relative to SPF mice. (**D**) Gene expression heatmap and pathway analyses for GMPs showing up- and down-regulation of genes in CVT relative to SPF mice. * *p* < 0.05.

**Figure A1:**
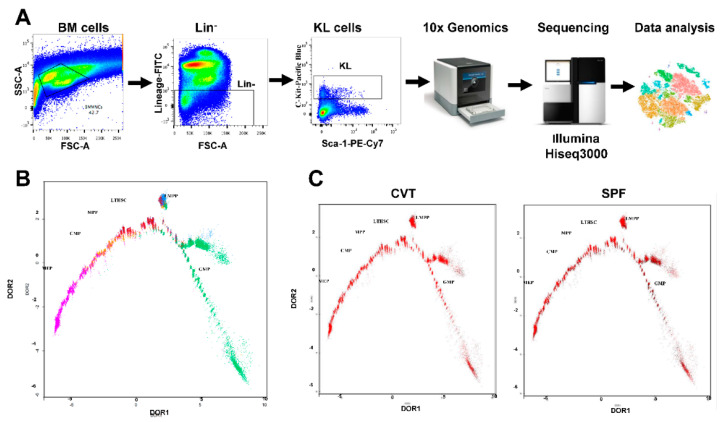
(**A**) Revelation of differential gene expression in Lin^−^Kit^+^ (KL) cells from CVT and SPF mice by single-cell RNA-seq. BM cells from 8 CVT and 8 SPF mice were sorted into KL cells, pooled to form 4 cell pools for each housing condition, and analyzed by single-cell RNA-seq. Illustration of cell sorting, genomics, sequencing and data analyses. (**B**) Gene expression defines KL cells into various subsets: LTHSC, long-term hematopoietic stem cells; MPP, Multipotent progenitors; CMP, common myeloid progenitors; MEP, megakaryocytic-erythroid progenitors; GMP, granulocyte-monocyte progenitors; LMPP, lymphoid-primed multipotent progenitors. (**C**) Hematopoietic cell subsets distribution in KL cells from CVT and SPF mice based on single cell RNA-seq.

Confirmation of a significant expansion in gut microbiota diversity in CVT mice led us to hypothesize that conventional co-housing might also affect gene expression and functional characteristics of HSPCs. We first performed single cell RNA-seq using sorted KL (c-Kit^+^Lin^−^) cells from BM of SPF and CVT mice at one month of co-housing (Figure A1A). We obtained high quality whole transcriptome data from ~30 × 10^3^ single KL cells which were clustered for CVT and SPF mice respectively based on unsupervised transcriptome similarity (Figure 3A). Hematopoietic cell identity was assigned to each cluster of cells by comparing cluster-specific genes with reported lineage signature genes [19], as reported previously [20]: KL cells were grouped into long-term hematopoietic stem cells (LTHSC), multipotent progenitors (MPP), lymphoid multipotent progenitors (LMPP), common myeloid progenitors (CMP), megakaryocyte-erythrocyte progenitors (MEP), and granulocyte-monocyte progenitors (GMP). While proportions of MPP, CMP, MEP and LMPP were similar for CVT and SPF mice, the proportion of LTHSC was lower and the proportion of GMP was higher in KL cells from CVT mice than those from SPF mice (Figure 3B). Pseudo-time temporal ordering was used to reconstruct hematopoiesis based on the transcriptomes of single KL cells (Figure A1B). Overall, co-housing did not alter the pattern of hierarchal hematopoiesis from multipotent stem cells to lineage-biased progenitors in CVT mice, nor did it affect the binary branching between megakaryocyte-erythroid progenitors and lymphoid and myeloid progenitors (Figure A1C).

**Figure A2:**
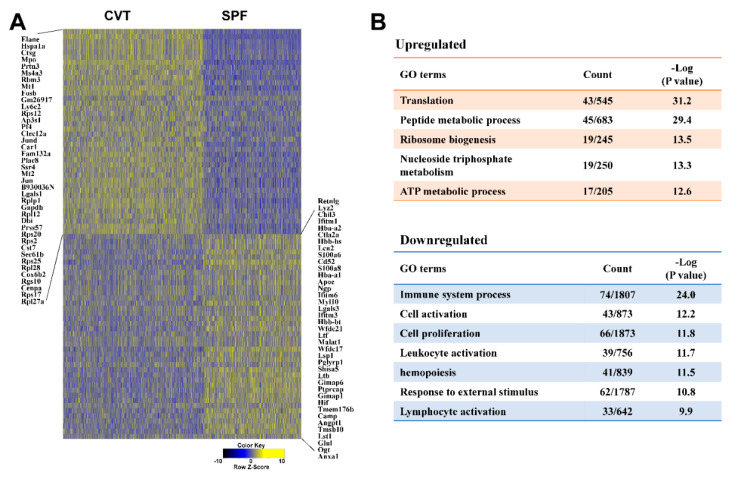
(**A**) Heatmap of gene expression shown distinctive patterns in KL (Lin^−^Kit^+^) cells from CVT and SPF mice following single-cell RNA-seq. (**B**) Pathway analyses showing up- and down-regulation of genes associated with signaling pathways in CVT relative to SPF mice.

Gene expression in KL, LTHSC and GMP cells showed 161, 240 and 2152 genes up-regulated and 288, 275 and 1640 genes down-regulated, respectively, in CVT mice relative to SPF mice. Table A2 lists the top 20 genes showing upregulation, while Table A3 lists the top 20 genes showing down regulation, in CVT mice relative to SPF mice in each of these three cell populations. In KL cells, patterns of gene expression differed significantly between CVT and SPF mice (Figure A2A, heatmap). Gene ontology (GO) pathway analyses revealed down-regulation of genes related to immune responses, cell proliferation and cell activation, and up-regulation of genes associated with cellular metabolism, including protein, ribosome, and energy metabolic processes, in cells from CVT mice (Figure A2B). In LTHSC, genes in the DNA damage-repair and genomic stability pathways were down-regulated whereas genes associated with cellular stimulation and cell survival pathways were up-regulated in CVT mice (Figure 3C). In GMP, genes involved in immune responses were down-regulated while genes associated with cell survival pathways were up-regulated in CVT mice (Figure 3D). In particular, we noted a significant up-regulation in the expression of ribosomal protein genes in GMP from CVT mice, relative to those from SPF mice (Table A2).

### 2.4. Functional Characteristics of HSPCs from CVT and SPF Mice

**Figure 4:**
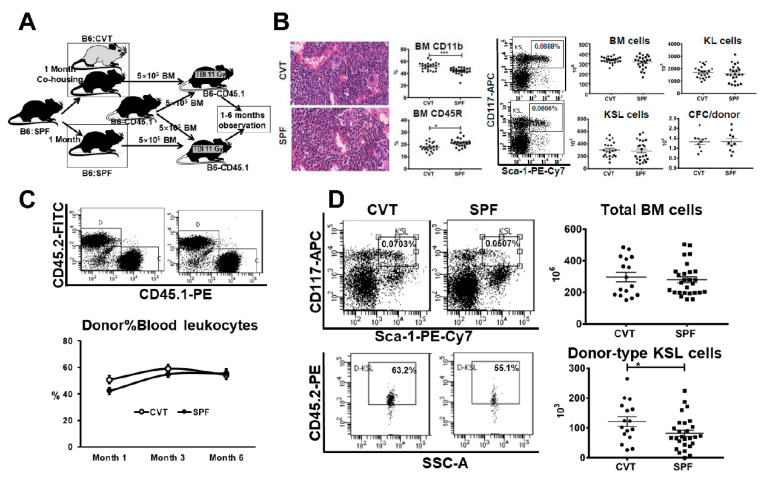
(**A**) C57BL/6J (B6) mice born and raised in specific-pathogen-free (SPF) conditions were either maintained in SPF or transferred to a conventional facility and co-housed (CVT) with mice born and raised in that facility for one month before they were euthanized and their bone marrow (BM) cells being transplanted into lethally-irradiated B6-CD45.1 recipients through competitive repopulation in vivo. (**B**) CVT (n = 22) and SPF (n = 23) mice were euthanized at one month after co-housing and representative sterna from each group were examined for BM histology, while BM cells from tibiae and femurs from all animals were stained and analyzed by flow cytometry for mature CD11b and CD45R cells and immature HSPCs carrying KL (c-Kit^+^Lin^−^) and KSL (c-Kit^+^Sca-1^+^Lin^−^) phenotypes. Recovery of total BM cells were used to calculate recovery of KL cells, KSL cells and colony forming cells (CFC) following eight days in methylcellulose culture. (**C**) BM cells from CVT (n = 10) and SPF (n = 10) donors were each mixed with BM cells from young B6-CD45.1 competitors and transplanted into lethally irradiated (11 Gys) B6-CD45.1 recipients through competitive repopulation at 2-3 recipients/donor. Recipients were bled at one, three and six months to assess donor contribution. (**D**) Competitive repopulation recipients were euthanized at six months after transplantation and recipient BM cells were stained and analyzed for the recovery of donor-type KSL cells. Data were combined from two separate competitive repopulation experiments. * *p* < 0.05.

Alterations in LTHSC and GMP proportions and changes in gene expression between CVT and SPF mice prompted a search for potential functional implications. We examined BM histology and analyzed BM cell composition in CVT and SPF mice and then mixed BM from CVT or SPF donors (CD45.2) 1:1 with BM cells from a pool of congenic B6-CD45.1 competitors and transplanted cell mixtures into lethally-irradiated B6-CD45.1 recipients to specifically test HSPC engraftment in vivo (Figure 4A). Examination of sterna BM found no major differences: all sterna had adequate cellularity at 90-5% levels with minimally increased overall myeloid/erythroid ratio in the CVT versus SPF donor mice (Figure 4B). BM cells flushed from tibiae and femurs were also not different in the recovery of total BM cells, KL cells, KSL (c-Kit^+^Sca-1^+^Lin^−^) cells and CFCs (colony forming cells) between CVT and SPF mice (Figure 4B). However, BM cells from CVT mice contained a higher proportion of CD11b myeloid cells and a lower proportion of CD45R B cells relative to those from SPF mice (Figure 4B). When BM cell were transplanted into lethally-irradiated recipients, overall mature blood cell engraftment was slightly higher in recipients of CVT donors at one and three, but not at six, months post transplantation: average donor contributions were 51%, 59% and 54% for recipients of CVT donors, and were 42%, 54% and 56% for recipients of SPF donors (Figure 4C). We euthanized recipient mice at six months after transplantation, and found, surprisingly, that BM cells from recipients of CVT donors contained higher proportions of KSL cells (0.07% vs. 0.05%, *p* < 0.01) and higher proportions of donor-derived KSL cells (0.05% vs. 0.03%, *p* < 0.01) than did BM cells from recipients of SPF donors, leading to significantly higher level recovery of donor-type KSL cells in recipients of CVT donors than in recipients of SPF donors (121 × 10^3^ vs. 82 × 10^3^, *p* < 0.05, Figure 4D). Thus, conventional co-housing produced enhanced donor KSL cell reconstitution but exerted minimal to no effect on recipient mature blood cell engraftment following BM transplantation.

### 2.5. Hematopoietic Effects Following Long-Term Co-Housing

**Figure 5:**
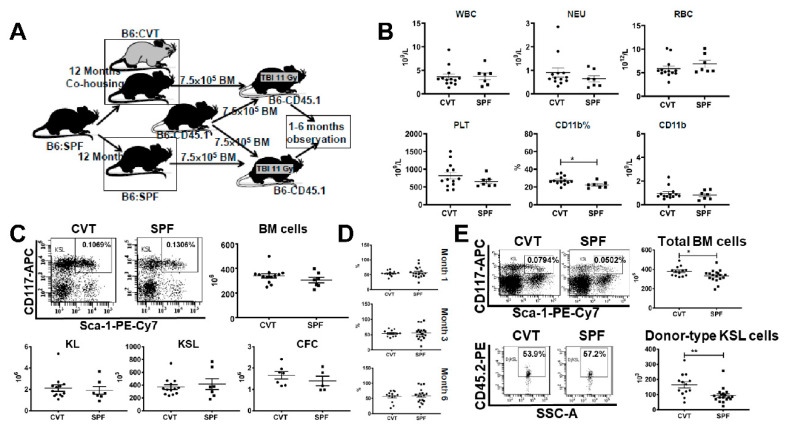
(**A**) B6 mice were maintained in SPF or were transferred to a conventional facility and co-housed (CVT) with mice from that facility for twelve months before they were euthanized and their BM cells being transplanted into lethally-irradiated (11 Gy) B6-CD45. 1 recipients in vivo through competitive repopulation. (**B**) Mice were bled at twelve months of co-housing in order to measure white blood cells (WBC), neutrophils (NEU), red blood cells (RBC) and platelets (PLT). The proportion and absolute number of blood CD11b myeloid cells were higher in CVT than in SPF mice. (**C**) BM cells from CVT and SPF donors were counted and analyzed to calculate recovery of total BM cells, KL (c-Kit^+^Lin^−^) cells, and KSL (c-Kit^+^Sca-1^+^Lin^−^) cells. BM cells from five CVT and five SPF donors were also cultured in vitro to measure colony forming cells (CFCs). No difference between CVT and SPF mice in these measurements. (**D**) Recipients of BM cells from CVT (n = 13) or SPF (n = 18) donors were bled and analyzed for donor contribution at six months following transplantation. (**E**) Recovery of total BM cells and donor-derived KSL cells in recipients of CVT and SPF donors at six months after BM transplantation. * *p* < 0.05; ** *p* < 0.01.

**Figure A3:**
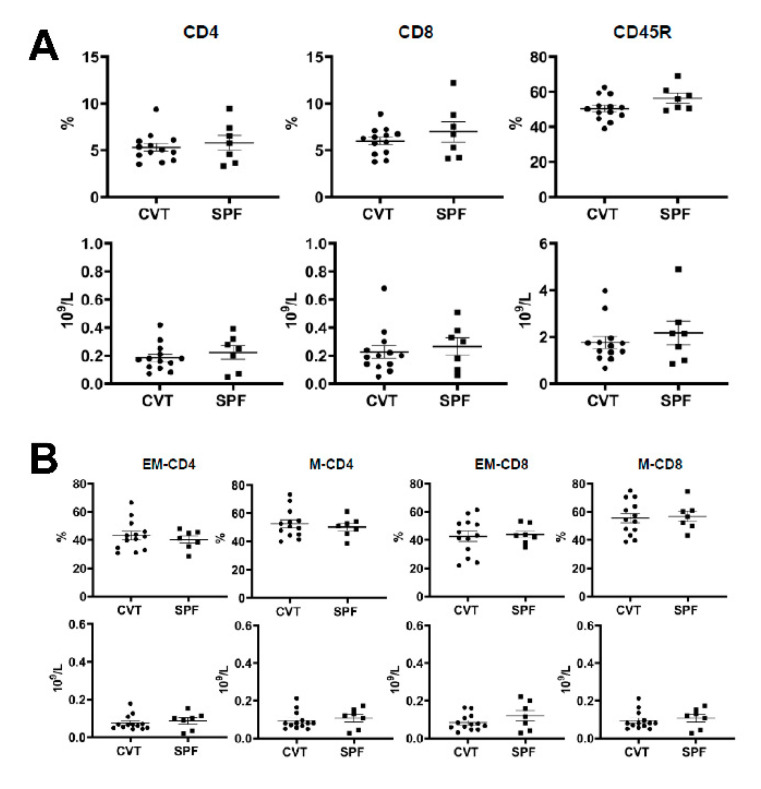
(**A**) B6 mice were maintained in SPF (N=7) or were transferred to a conventional facility and co-housed (CVT, N = 13)) with mice from that facility for twelve months. Mice were bled at twelve months of co-housing to analyze proportions and absolute numbers of CD4, CD8 and CD45R lymphocytes in peripheral blood. (**B**) CD4 and CD8 T cells were further divided into EM-CD4, M-CD4, EM-CD8 and M-CD8 subsets based on CD44 and CD62L expression in which proportions and absolute numbers of EM-CD4, M-CD4, EM-CD8 and M-CD8 were similar for CVT and SPF mice.

We extended co-housing in the conventional facility to twelve months and measured blood and bone marrow cell numbers (Figure 5A). There were no difference in WBC, NEU, RBC and platelet concentrations (Figure 5B), and no difference in the proportions and absolute numbers of blood CD4, CD8 and CD45R (Figure A3A). When CD4 and CD8 T cells were further divided into different subsets based on CD44 and CD62L expression, we found no difference in the proportions and concentrations of EM-CD4, M-CD4, EM-CD8 and M-CD8 cells between CVT and SPF mice (Figure A3B). However, the proportion (27.5% vs. 22.4%, *p* < 0.05) and absolute numbers (0.95 × 10^9^/L vs. 0.82 × 10^9^/L) of CD11b myeloid cells were higher in CVT than in SPF mice (Figure 5B). Recovery of total BM cells, KL cells, KSL cells and CFCs were also not different between CVT and SPF mice after twelve months of co-housing (Figure 5C). Transplantation of BM cells from CVT and SPF donors into lethally-irradiated B6-CD45.1 recipients through competitive repopulation showed no significant difference in mature blood cell engraftment at one, three and six months after transplantation (Figure 5D). However, recipients of CVT donors contained significantly more total BM cells (379 × 10^6^ vs. 332 × 10^6^, *p* < 0.05) and more donor-type KSL cells (161 × 10^3^ vs. 93 × 10^3^, *p* < 0.01) than in recipients of SPF donors, at six months after transplantation (Figure 5E). Thus, after twelve months of co-housing, CVT mice maintained a higher level of blood CD11b myeloid cells and a higher level of donor KSL cell reconstitution, but without a corresponding gain in mature blood cell engraftment, relative to age-matched SPF mice.

## 3. Discussion

In the current study, we adapted a method of co-housing SPF mice with “dirty” mice [1], and compared SPF mice with CVT mice that were co-housed with CVB mice in a conventional facility for one or twelve months. Findings from our study were in general agreement with earlier reports comparing SPF mice with GF mice [6,12], or comparing SPF mice with or without antibiotic treatment [6,7,13], showing enhancements in hematopoietic and immune characteristics with increasing gut microbiota diversity [1,2]. In particular, the higher proportions of effector memory and memory CD4 and CD8 T cells in the blood of CVT mice are in concordance with observations from pet-store co-housed laboratory mice [1]. Changes in these immune phenotypes were less dramatic in the CVT mice in the current study than in pet-store co-housed mice reported earlier, which is consistent with a smaller change in microbial diversity in conventionally-house mice than in pet-store mice. An enlarged population of CD11b myeloid cells is another phenotype we observed in CVT mice, possibly indicative of augmented immune function following a gain in gut microbiota diversity, and consistent with more CD11b myeloid cells in SPF than in GF mice [6], showing that gut microbial stimulate CD11b myeloid cell production to enhance innate immunity.

Analysis of gut microorganisms revealed significant differences between CVT and SPF mice showing effective transfer of microorganisms from CVB to CVT mice through co-housing. The top three OTUs highly represented in CVB mice and effectively transferred to CVT mice during co-housing were *Prevotellaceae, Lactobacillaceae* and *Rikenellaceae*, which is consistent with our previous observation in CByB6F1 mice born and housed in the same conventional facility in which the same three microbial families were most abundant in CVB mice [17].These microbial families offer health benefits, since lack of *Prevotellaceae* and *Rikenellaceae* have been associated with Parkinson’s disease [21], and liver cirrhosis [22]. In Rag1-/- mice, fecal transplants from wild type donors altered gut bacterial community with increases in *Prevotellaceae* which were accompanied by increases in total number of BM cells and HSPCs [23]. By contrast, the presence of certain *Prevotella* species have been associated with increased risk for myeloma, perhaps by promoting the differentiation of Th17 cells in the gut which migrate to the BM [24]. We recognize that co-housing as in the current study encompasses multiple potentially confounding factors: food source and processing, ventilation and cage covering, as well as facility barrier and access control, which may contribute to the differences we observed between SPF and CVT mice. Our use of the term “conventional co-housing” represents the sum of these contributing factors, and each or several could be further explored in future studies.

Reduction in LTHSC and increase in GMP in CVT mice were fresh observations from our study that demonstrate a potential association between gut microbiota diversity and hematopoiesis regulation. Single cell RNA-seq enabled us to divide HSPCs into functional subsets based on global gene expression rather than on a few cell surface markers [20]. The decrease in LTHSC and the increase in GMP in CVT mice are likely correlated, as well as to the increase in CD11b myeloid cells, as general responses to stimulation from a more diversified microbiota augmenting hematopoietic differentiation favoring the myeloid lineage. Increase in GMP in CVT mice may help to explain the small gain in CVT donor cell engraftment to recipient mature blood cells at one and three months after transplantation.

A unique observation from this study was an increase in KSL cell reconstitution from CVT donors after both short- and long-term co-housing. The gain of donor KSL cell reconstitution could be the result of an increased response of CVT donor BM cells in the conventional environment that increases the KSL pool size. Administration of the inflammatory cytokine gamma interferon stimulates Sca-1 expression and can significantly increase KSL pool size [25,26,27]. However, we did not observe a gain of KSL cells in CVT mice even after long-term co-housing, thus it is unlikely that the conventional housing environment stimulates Sca-1 expression to artificially increase an apparent KSL pool size. Furthermore, enhanced KSL cell reconstitution in our study was not associated with much decline in mature blood cell engraftment, as was seen in mice following gamma interferon treatment [26], indicating that the increased donor KSL cells in recipients that received CVT BM cells are functionally active. It is possible that the gain of KSL cell reconstitution from CVT donors could be the result of increased KSL cell survival, since expression of genes associated with cell survival was increased in LTHSC and GMP cells from CVT mice. Upregulation of genes associated with cell survival pathways, in the TLR and Wnt signaling pathways, may contribute to the enhanced KSL cell reconstitution phenotype from CVT donors. Engagement of TLR ligands with TLRs regulate HSC differentiation and lineage specification [28,29], activation of canonical Wnt-β-catenin signaling in combination with mTOR suppression maintains human and mouse LT-HSC ex vivo under cytokine-free conditions [30], and up-regulation of canonical and non-canonical Wnt signaling pathways can led to increases in lymphopoiesis and myelopoiesis [31]. We posit that molecular changes in LTHSC and GMP could provide a survival advantage in CVT KSL cells, leading to a gain in CVT-donor-KSL cell reconstitution relative to the reconstitution of competitor-originated KSL cells. The survival advantage would not be observed in the absence of competitor KSL cells, and indeed no difference in the size of the KSL cell pool was seen between CVT and SPF mice at steady state, without BM transplantation.

The gain in KSL reconstitution from CVT donors was not associated with a corresponding improvement in donor engraftment. It is possible that the expanded KSL cells from CVT donors may have reduced functional capacity per cell or that some of the expanded KSL cells from CVT donors are quiescent and do not contribute to recipient mature blood cell production. Future studies are needed to discriminate among these possibilities.

## 4. Materials and Methods

### 4.1. Mice and Animal Husbandry

Inbred B6 mice were initially obtained from the Jackson Laboratory (Bar Harbor, ME, USA) and maintained for three weeks in a barrier-controlled SPF facility with covered cages, individual cage air ventilation and free access to autoclaved 75 IF 5V0A rodent chow (Purina LabDiet, St. Louis, MO, USA). Mice were then split into two groups: SPF mice were maintained in the SPF facility while CVT mice were transferred from SPF to a conventional facility and co-housed with CVB mice (four transferred SPF mice housed in the same cage with one CVB mouse) for one or twelve months. The conventional facility has a limited barrier control in which mice were housed in open-top cages on suspended shelves with free access to unautoclaved 5001c Rodent chow (Purina LabDiet, St. Louis, MO, USA). Congenic B6-CD45.1 (B6.SJL-Ptprc^a^Pep3^b^/Boy) mice, also from the Jackson Laboratory, were maintained in SPF and conventional facilities as BM recipients. Animals in both facilities had free access to acidified water at pH 2.7-3.0. All animal studies were approved by Institutional Animal Care and Use Committees at National Heart, Lung, and Blood Institute and the National Cancer Institute, respectively.

### 4.2. Cell Counting, Histology and Flow Cytometry

Blood was collected from retro-orbital sinus into eppendorf tubes in the presence of 5 mM ethylenediaminetetraacetic acid (EDTA, Sigma, St. Louis, MO, USA). Complete blood counts (CBC) were performed using a HemaVet 950 analyzer (Drew Scientific, Inc., Miami Lake, FL, USA). After euthanasia, sterna were collected from CVT and SPF mice, fixed in 10% neutral buffered formalin, sectioned at 5 µM thickness, and stained with hematoxylin and eosin (VitroVivo Biotech, LLC., Rockville, MD, USA). Slides were examined under a Zeiss Axioskop 2 microscope (Carl Zeiss MicroImaging GmbH, Jena, Germany) and images were captured at 20× magnification. BM cells were extracted from bilateral tibiae and femurs into RPMI 1640 media (Invitrogen, Carlsbad, CA, USA) supplemented with 10% fetal bovine serum, 100 U/mL penicillin, 100 μg/mL streptomycin, and 100 μg/mL glutamine), filtered through 90 μM nylon mesh (Small Parts, Miami Lake, FL, USA), and counted by a Vicell counter (Beckman Coulter, Miami, FL, USA).

BM and blood cells were stained with antibody mixtures for flow cytometry analyses. Monoclonal antibodies for murine CD3e (clone 145-2C11), CD4 (Clone GK1.5), CD8a (Clone 53-6.7), CD11b (clone M1/70), CD44 (clone 1M7), CD45R (clone RA3-6B2), CD45.1 (Clone A20), CD45.2 (clone 104), CD62L (MEL-14), CD117 (Clone 2B8), erythroid cells (Clone TER-119), granulocytes (Clone RB6-8C5), and stem cell antigen 1 (Sca1, Clone D7) were all from Biolegend (San Diego, CA, USA) and were conjugated to fluorescein isothiocyanate (FITC), phycoerythrin (PE), PE-cyanin 5 (PE-Cy5), PE-cyanin 7 (PE-Cy7), allophycocyanin (APC), APC-cyanin 7 (APC-Cy7) or brilliant violet 421 (BV421). Stained cells were analyzed in a Canto II flow cytometer with data acquisition and analyses by FACSDiva software (Becton Dickson, San Diego, CA, USA). We use KL (c-Kit^+^Lin^−^) and KSL (c-Kit^+^Sca-1^+^Lin^−^) as phenotypic markers for HSPCs.

### 4.3. 16S rRNA Gene Amplicon Sequencing and Metagenomic Analyses

Mouse fecal pellets were collected from CVB, CVT and SPF animals before and one to twelve months after co-housing in the conventional facility. Fecal samples were stored at −80 °C and were loaded into a 96 well plate when ready for analysis. DNA extraction and amplification were performed using Eppendorf liquid handling robots. The V4 region of the 16 rRNA (515F-806R) was amplified and sequenced generating paired-end, overlapping reads using the Illumina MiSeq platform [32]. The demultiplexed paired-end FASTQ files were pre-processed and analyzed using QIIME2 version 2.2017.8 [33]. The DADA2 algorithm [34], implemented in QIIME2, was used for error modelling and filtering the raw FASTQ files. Post de-noising and chimera removal; a total of 2,521,892 sequences were retained for 39 samples with an average of 64,663 sequences per sample. Taxonomic classification was performed using the QIIME2 feature-classifier plugin trained on the Silva 132 database [35]. Alpha and Beta-diversity analyses were performed using the diversity plugin at a sampling depth of 28,892 sequences. Principal component analysis plots were generated using Emperor [36], and Linear Discriminant Analysis was performed using LefSe [37]. Shotgun metagenomic data was generated on the Illumina NextSeq platform. Bioinformatic analyses were performed using an in-house package in R; YAMS version 0.960T (https://github.com/johnmcculloch/JAMS_BW) [3].

### 4.4. Single Cell RNA Sequencing

BM cells from CVT and SPF mice at one month of co-housing were stained and sorted to collect KL cells (Figure A1A) using a FACSaire cell sorter (Beckton Dickson). Single cell transcriptome analysis was conducted using the Chromium Single-Cell 3′ Reagent v2 Kit (10× Genomics, Pleasanton, CA, USA) as per the manufacturer’s protocol. Single cell RNA-seq libraries were sequenced on an Illumina HiSeq 3000 System (Illumina, San Diego, CA, USA) with a customized paired end, single indexing (26/8/0/98-bp) format according to 10X Genomics recommendations. Alignment, barcode assignment and UMI counting were performed using the Cell Ranger pipeline [38]. In total, 12,392 and 17,629 KL single cell libraries were captured from SPF and CVT mice respectively, with 10 × 10^4^ and 8 × 10^4^ mean reads per cell and 2.5 × 10^3^ medium genes detected per cell. Genes with at least one UMI count detected in at least one cell were used (filtering data). The top 1000 most variable genes were identified based on their mean and dispersion (variance/mean). Twenty-three graph-based clusters of cells were classified by two-dimensional t-distributed Stochastic Neighbor Embedding (tSNE) using Seurat2 (resolution 2). In each cluster, the mean expression of each gene was calculated across all cells to identify genes that were enriched in a specific cluster. Each gene from the cluster was compared to the median expression of the same gene from cells in all other clusters. Genes were ranked based on their expression differences, and the top enriched genes were compared with reported gene differential analysis using Seurat 2 [39,40]. Cells were mapped and clustered using tSNE and DBSCAN on highly variant genes [39]. HSPC subtype was assigned to each cluster based on overlap significance between HSPC- and cluster-specific genes (Fisher’s exact test). Single cell gene expression data was been submitted to National Center for Biotechnology Information Gene Expression Omnibus (GEO accession number: GSE142235).

### 4.5. Competitive Repopulation and Colony Forming Cell (CFC) Assay

BM cells from SPF donors, or from CVT donors at one or twelve months of co-housing, were mixed 1:1 with BM cells from a pool of congenic B6-CD45.1 competitors, and the cell mixtures were then injected into B6-CD45.1 recipients pre-irradiated with 11 Gy total body irradiation (TBI, from a Shepherd Mark 1^137^Cesium gamma source, J. L. Shepherd & Associates, Glendale, CA, USA) 4–6 h earlier at 10^6^ cells per recipient, half donor and half competitor. Recipients were bled at one, three and six months after transplantation, and blood samples were stained for flow cytometry analyses to measure donor contribution. Recipient mice were euthanized six months after transplantation and BM cells were extracted from tibiae and femurs, counted, and stained for flow cytometry analyses to measure donor KSL cell reconstitution.

BM cells from SPF and CVT mice at one and twelve months of co-housing were also cultured in semisolid methylcellulose media containing interleukin-3, interleukin-6, stem cell factor and erythropoietin (Stem Cell Technology Inc, Vancouver, BC, Canada) at 3 × 10^4^ cells/mL under 37 °C with 5% CO_2_. Colonies were counted at day eight.

### 4.6. Statistics

Data from blood and BM cell counts and from flow cytometry were analyzed by unpaired t test using GraphPad Prism statistical software (GraphPad Software, Inc., La Jolla, CA, USA), or by two-way ANOVA with JMP statistical discovery software (SAS Institute Inc., Cary, NC, USA), and were presented as means with standard errors. Statistically significant differences were marked as: * *p* < 0.05; ** *p* < 0.01; *** *p* < 0.001 and **** *p* < 0.0001.

## 5. Conclusions

Co-housing laboratory mice with conventionally-housed mice increased gut microbial biodiversity, elevated circulating myeloid cells and T cells especially activated effort memory T cells, promoted LTHSC differentiation to increase GMP, augmented expression of genes regulating cell survival, and enhanced KSL cell reconstitution. These effects did not yield a significant gain in the engraftment of recipient mature blood cells when tested by competitive repopulation.

## Abbreviations

APC	allophycocyanin
B6	C57BL/6
BM	Bone marrow
CFC	Colony forming cells
CMP	Common myeloid progenitors
CVB	Mice born and raised in conventional facility
CVT	SPF mice transferred and co-housed in conventional facility
EM	Effector memory
GMP	Granulocyte-monocyte progenitors
HSPC	Hematopoietic stem and progenitor cells
KL	c-Kit^+^Lin^−^ cells
KSL	c-Kit^+^Sca-1^+^Lin^−^ cells
LMPP	Lymphoid multipotent progenitors
LTHSC	Long-term hematopoietic stem cells
MEP	Megakaryocyte-erythrocyte progenitors
MPP	Multipotent progenitors
OTU	operational taxonomic units
PE	phycoerythrin
SPF	Specific-pathogen-free
tSNE	t-distributed stochastic neighbor embedding

## Appendix A

**Table A1:** Differential representation of bacterial strains in fecal samples from CVT and SPF mice.

Rank	Bacterial Name	CVT	SPF	Main Characteristics
01	Bacteroides mediterraneensis	H	L	G-, anaerobic, rod-shared, isolated in 2016
02	Bacteroides stercoris	H	L	Symbiont, abundant, opportunistic pathogens
03	Prolixibacter denitrificans	H	L	G-, anaerobic, nitrate-reducing, isolated in 2015
04	Butyricimnas synergistica	H	L	G- anaerobic, rod-shaped, new genus found in 2009
05	Lactobacillus reuteri	H	L	Antimicrobial, reduce pro-inflammatory cytokines
06	Prevotella multiformis	H	L	G-, cocci and rods, anaerobic, isolated in 2005
07	Prevotella lascolaii	H	L	G-, anaerobic, rod-shaped, isolated in 2018
08	Oligotropha carboxidovorans	H	L	G-, growth on CO, CO2 and H2, reported in 2013
09	Bacillus campisalis	H	L	G+, isolated from solar saltern samples in 2015
10	Paraprevotella xylaniphila	H	L	G-. anaerobic rods, isolated in 2009
11	Sediminispirochaeta smaragdinae	H	L	Anaerobic, spiral, reduce thiosulfate, isolated in 1998
12	Prevotella melaninogenica	H/L	L	G-, anaerobic, rod, classified in 1921, 1982 and 1990
13	Chryseobacterium gleum	L	H	G-, aerobic, bacilli, yellow, respiratory infection
14	Psedomonas Stutzeri	L	H	G-, aerobic, rod-shaped, opportunistic pathogen
15	Parabacteroides goldsteinii	H	L	G-, anaerobic, pathogenic for abdominal infections

H: high level representation; L: low level representation.

**Table A2:** Genes up-regulated in KL, LTHSC, and GMP hematopoietic cells from CVT mice at one month after co-housing relative to SPF mice.

	KL			LTHSC			GMP	
Gene Name	P	LogFold	Gene Name	P	LogFold	Gene Name	P	LogFold
Elane	0.0000	0.3628	S100a8	0.0000	1.0331	Hspa1a	0.0000	0.3417
Hspa1a	0.0000	0.3340	S100a9	0.0000	0.9907	Rpl12	0.0000	0.2177
Ctsg	0.0000	0.3111	Camp	0.0000	0.9549	Rps12	0.0000	0.2167
Mpo	0.0000	0.2878	Ngp	0.0000	0.7470	Rplp1	0.0000	0.2027
Prtn3	0.0000	0.2591	Car1	0.0004	0.5737	Rbm3	0.0000	0.2012
Ms4a3	0.0000	0.2247	Lcn2	0.0000	0.5150	Jun	0.0000	0.1942
Mt1	0.0000	0.1746	Klf6	0.0001	0.3092	Ctsg	0.0000	0.1874
Fosb	0.0000	0.1696	Mpo	0.0043	0.2795	Rps2	0.0000	0.1847
Gm26917	0.0000	0.1580	Mt1	0.0253	0.2753	Prtn3	0.0000	0.1826
Ly6c2	0.0000	0.1483	Atf4	0.0002	0.2687	Rpl10a	0.0000	0.1812
Rps12	0.0000	0.1442	Rbm3	0.0001	0.2601	Eefla1	0.0000	0.1771
Ap3S1	0.0000	0.1397	Ly6c2	0.0011	0.2546	Eeflb2	0.0000	0.1746
Pf4	0.0000	0.1291	Jund	0.0155	0.2490	Rpl14	0.0000	0.1736
Clec12a	0.0000	0.1290	Fos	0.0059	0.2440	Hsp90ab1	0.0000	0.1697
Jund	0.0000	0.1277	Dusp1	0.0000	0.2374	Gm26917	0.0000	0.1674
Car1	0.0000	0.1255	Klf2	0.0118	0.2335	Mpo	0.0000	0.1657
Fam132a	0.0000	0.1244	Dusp1	0.0065	0.2147	Elane	0.0000	0.1651
Plac8	0.0000	0.1240	Ik	0.0126	0.2101	Rpsa	0.0000	0.1641
Ssr4	0.0000	0.1191	Serpinb6a	0.0017	0.2096	Rpl27a	0.0000	0.1640
Mt2	0.0000	0.1170	Fam132a	0.0448	0.1978	Zfos1	0.0000	0.1635

Data shown as top 20 genes up-regulated in CVT mice.

**Table A3:** Genes down-regulated in KL, LTHSC, and GMP hematopoietic cells from CVT mice at one month after co-housing relative to SPF mice.

	KL			LTHSC			GMP	
Gene Name	P	LogFold	Gene Name	P	LogFold	Gene Name	P	LogFold
Retnlg	0.0000	−0.5532	Mien	0.0001	−0.2787	Retnlg	0.0000	−0.9036
Lyz2	0.0000	−0.3917	Nop56	0.0030	−0.2564	Lyz2	0.0000	−0.6427
Chil3	0.0000	−0.2833	Ninj1	0.0247	−0.2253	Hbb−bs	0.0000	−0.5974
Ifitm1	0.0000	−0.2744	Cstb	0.0045	−0.2203	Chil3	0.0000	−0.5521
Hba-a2	0.0000	−0.2507	Chchd6	0.0014	−0.2194	S100a6	0.0000	−0.4957
Ctla2a	0.0000	−0.2449	Snx5	0.0056	−0.2192	Lcn2	0.0000	−0.4640
Hbb-bs	0.0000	−0.2332	Dapp1	0.0174	−0.2173	Ifitm6	0.0000	−0.4616
Cd52	0.0000	−0.2050	Ivnslabp	0.0033	−0.2168	S100a8	0.0000	−0.4468
S100a9	0.0061	−0.2012	Eef2k	0.0024	−0.2165	S100a9	0.0000	−0.4446
Hba-a1	0.0018	−0.1960	Sdpr	0.0012	−0.2144	Lgals3	0.0000	−0.4394
Apoe	0.0000	−0.1891	Timm22	0.0032	−0.2109	Ngp	0.0000	−0.4284
Ngp	0.0080	−0.1880	Vps29	0.0029	−0.2088	Cd52	0.0000	−0.4141
Ifitm6	0.0487	−0.1850	Serpinb1a	0.0411	−0.2061	Mmp8	0.0000	−0.4118
Myl10	0.0000	−0.1793	Mycbp2	0.0154	−0.2050	Wfdc21	0.0000	−0.4080
Lgals3	0.0000	−0.1728	Gpn3	0.0020	−0.2021	Ltf	0.0000	−0.4076
Ifitm3	0.0000	−0.1706	Hif1a	0.0013	−0.1985	Ccl6	0.0000	−0.3996
Hbb-bt	0.0000	−0.1638	Hnrnph3	0.0157	−0.1984	Pgiyrp1	0.0000	−0.3930
Malat1	0.0000	−0.1565	Kansl11	0.0001	−0.1969	Camp	0.0000	−0.3696
Wfdc17	0.0000	−0.1487	Vezf1	0.0064	−0.1948	Ly6g	0.0000	−0.3681
Lsp1	0.0000	−0.1434	Dhx36	0.0255	−0.1936	Hba−a1	0.0000	−0.3539

Data shown as top 20 genes down-regulated in CVT mice.
